# Association Between Intravenous Magnesium Sulfate and All-Cause Mortality in Patients With Acute Heart Failure: A Propensity Score-Matched Cohort Study in MIMIC-IV

**DOI:** 10.31083/RCM39206

**Published:** 2025-09-24

**Authors:** Lin Na, Jing Chang, Xinqi Li, Xiaona Che, Yunfei Sun, Wenjing Cui, Wenhao Zhang, Xin Xue

**Affiliations:** ^1^Department of Cardiology, The Second Hospital of Jilin University, 130041 Changchun, Jilin, China; ^2^Clinical Laboratory, The Second Hospital of Jilin University, 130041 Changchun, Jilin, China; ^3^Department of Cardiology, Xi'an International Medical Center Hospital, 710100 Xi'an, Shaanxi, China

**Keywords:** intravenous, magnesium sulfate, acute heart failure, all-cause mortality

## Abstract

**Background:**

Magnesium deficiency represents a prevalent electrolyte imbalance that notably heightens the risk of adverse cardiovascular incidents among individuals with heart failure. Research indicates that magnesium supplementation can diminish the frequency of negative events linked to hypomagnesemia in heart failure patients, while also enhancing the outcomes for critically ill individuals. Nevertheless, there remains a scarcity of studies investigating the effects of intravenous magnesium supplementation on mortality risk in those suffering from acute heart failure. This study aimed to explore the relationship between the administration of intravenous magnesium sulfate and the overall mortality risk in patients with acute heart failure who are admitted to intensive care units.

**Methods:**

This retrospective cohort study employed the Medical Information Mart for Intensive Care IV (MIMIC-IV) database (version 3.1), which encompasses the baseline clinical data of 10,031 patients diagnosed with acute heart failure. Propensity score matching and multivariate Cox regression analysis were utilized to assess the effects of intravenous magnesium sulfate on 28-day all-cause mortality. The evaluation of unmeasured confounding variables was undertaken through E-value calculations, while subgroup analyses were performed to ascertain the robustness of the results.

**Results:**

A total of 10,031 patients participated in the study, with 6240 belonging to the propensity-score matched group, which included 3120 subjects who received intravenous magnesium sulfate and 3120 who did not. The entire cohort consisted of 10,031 individuals, whereas the matched cohort specifically comprised 6240 patients. Within the matched group, the rates of 28-day all-cause mortality were observed to be 13.2% (413/3120) for those administered intravenous magnesium sulfate, compared to 15.8% (493/3120) for the control group. Administration of intravenous magnesium sulfate correlated with a significantly reduced risk of 28-day all-cause mortality (hazard ratio (HR), 0.81; 95% confidence interval (CI), 0.71–0.93; *p *= 0.004). A sensitivity analysis conducted on the entire cohort corroborated this association (HR, 0.77; 95% CI, 0.69–0.87; *p *< 0.001). Additional subgroup analyses and E-value assessments reinforced the relevance of these results. Furthermore, intravenous magnesium sulfate was linked to reduced all-cause mortality at both 90 and 365 days.

**Conclusions:**

Intravenous magnesium sulfate appears to decrease all-cause mortality in patients with acute heart failure, while further prospective studies are warranted to validate these results.

## 1. Introduction

Acute heart failure (AHF) represents a critical global health challenge, associated with extremely high short and long-term mortality rates [1]. The in-hospital mortality rate ranges from 4% to 12%, while the 1-year mortality rate post-discharge can be as high as 21% to 35% [2, 3]. The primary causes of death include cardiogenic shock and multiple organ failure. This condition not only triggers severe symptoms such as dyspnea and systemic hypoperfusion but also leads to multiple organ dysfunction, significantly reducing quality of life and survival. The associated healthcare burden is substantial, accounting for approximately 2% of total national healthcare expenditures in high-income countries [4].

Magnesium, following potassium, ranks as the second most prevalent intracellular cation and is essential for the functioning of the cardiovascular system [5, 6]. It plays a crucial role in regulating neuronal excitation, cardiac conduction, and myocardial contraction by influencing various ion transporters, including both potassium and calcium channels [7]. A common issue among patients with heart failure is magnesium deficiency [8], which can worsen hemodynamic instability and elevate the likelihood of ventricular arrhythmias, thus becoming a major risk element for adverse cardiovascular events within this group [8, 9, 10, 11, 12, 13, 14]. Intravenous magnesium supplementation has emerged as a potential adjunctive strategy due to its dual roles: ① counteracting neurohormonal overactivation by modulating calcium channels, and ② improving cardiac contractility through its antioxidant effects. Magnesium supplementation has been shown to mitigate the adverse events associated with hypomagnesemia in heart failure patients and improve outcomes in critically ill patients [10, 14, 15].

Nonetheless, evidence concerning the effect of intravenous magnesium supplementation on mortality rates in patients suffering from acute heart failure is scarce, indicating a need for additional research. In this research, we employed the Medical Information Mart for Intensive Care IV (MIMIC-IV) database to explore the link between the administration of intravenous magnesium sulfate and mortality among individuals with acute heart failure.

## 2. Materials and Methods

### 2.1 Data Source

Our cohort investigation employed a retrospective design that matched subjects using propensity scores, utilizing the MIMIC-IV electronic database (version 3.1) as its foundation. A comprehensive list of patients from MIMIC-IV was assembled, encompassing all medical record numbers linked to individuals admitted to either the intensive care unit (ICU) or the emergency department from 2008 to 2022. This included a total of 546,028 hospitalizations and 94,458 distinct stays in the ICU at Beth Israel Deaconess Medical Center located in Boston, MA, USA. The corresponding author (Xin Xue) successfully completed the exam for the Collaborative Institutional Training Initiative (CITI) program and received a certificate (Record ID: 53098597). The research adhered to the ethical principles set forth in the Declaration of Helsinki and complied with the Reporting of Observational Studies in Epidemiology (STROBE) guidelines [16]. Given that the data utilized in this clinical research were sourced from public databases, the Ethics Review Committee at the Second Hospital of Jilin University classified it as exempt from an ethical review.

### 2.2 Study Participants

Acute heart failure was defined by pulmonary congestion, systemic congestion, and inadequate perfusion of tissues and organs. It was categorized into four primary types: acute decompensated heart failure, acute pulmonary edema, cardiogenic shock, and isolated right ventricular failure.

In the MIMIC-IV database, patients meeting inclusion criteria for AHF were identified based on the International Classification of Diseases 9th edition (ICD-9) codes (42833, 42823, 42843, 42831, 42821, 42841) and the ICD-10 codes (I5033, I5023, I5021, I5031, I5043, I5041). Patients under 18 years of age, those who had been given magnesium sulfate prior to ICU admission, or instances where blood magnesium data was unavailable were excluded from the study. Furthermore, we limited our analysis to the initial admission records of patients who had multiple ICU admissions.

### 2.3 Exposure and Outcomes

The use of intravenous magnesium sulfate during the ICU stay was the exposure being examined, without any restrictions. Data concerning the administration of magnesium sulfate were gathered from the prescription table. The main outcome measured was mortality from any cause within 28 days. Additionally, secondary outcomes encompassed mortality from any cause at 90 days and at 365 days.

### 2.4 Data Extraction

Data was extracted utilizing Structured Query Language (SQL) across five distinct categories: (1) Demographics, which encompassed age, gender, race, and types of intensive care units (ICUs); (2) Physical assessments and laboratory tests, detailing heart rate, systolic blood pressure (SBP), diastolic blood pressure (DBP), mean arterial pressure (MAP), respiratory rate, body temperature, and magnesium status at the onset of acute heart failure (including hypomagnesemia, hypermagnesemia, and normomagnesemia), along with white blood cell count (WBC), platelet count, glucose levels, creatinine levels, magnesium levels, potassium levels, sodium levels, calcium levels, chloride levels, phosphate levels, albumin, aspartate aminotransferase, and alanine aminotransferase levels; (3) Comorbidities, which included QT prolongation syndromes, hypertension, diabetes, myocardial infarction, cerebrovascular diseases, peripheral vascular diseases, dementia, chronic pulmonary diseases, renal disease, severe liver disease, rheumatic diseases and malignant cancer; (4) Special treatments, consisting of mechanical ventilation, intravenous diuretics (furosemide), vasodilator (sodium nitroprusside or nitroglycerin), vasopressor (norepinephrine, dopamine or dobutamine), digoxin, statins, angiotensin converting enzyme inhibitor (ACEI), angiotensin II receptor blockers (ARB), aspirin, β-blockers, and adenosine diphosphate (ADP) receptor antagonists; (5) Clinical scores, including Charlson comorbidity index, Acute Physiology and Chronic Health Evaluation Ⅲ (APACHE Ⅲ), Simplified Acute Physiology Score Ⅱ (SAPS Ⅱ) and Oxford acute severity of illness score (OASIS).

### 2.5 Statistical Analysis

This research utilized a retrospective approach, dividing participants into two categories: the group that received magnesium sulfate (designated as the magnesium sulfate group) and the group that did not receive it (referred to as the no-magnesium sulfate group). The missing values for each variable were estimated through multiple imputation methods. The presence of multicollinearity among covariates was evaluated by examining the variance inflation factor (>10) and tolerance values (>0.1).

Data that demonstrated a normal distribution, as verified by the Kolmogorov-Smirnov test, were reported as mean ± standard deviation; a *t*-test was conducted to assess differences between the two groups. For data exhibiting skewed distributions, results were shown as median (interquartile range), with group comparisons performed using a rank sum test. Count data were indicated by the number of cases (n) and their corresponding percentages (%), analyzed through the chi-square test. A multivariate cox proportional hazards model was constructed to evaluate the hazard ratio (HR) and its associated 95% confidence interval (CI) concerning magnesium sulfate treatment and the risk of all-cause mortality in patients with acute heart failure. The Kaplan–Meier approach was employed to study the cumulative incidence of 28-day all-cause mortality, with comparisons made through the log-rank test. For secondary outcomes, HR and 95% CI were determined using multivariate cox regression models.

#### 2.5.1 Propensity Score Matching

In the matched cohort, we conducted primary analyses to explore the relationship between the use of magnesium sulfate and both primary and secondary outcomes. Propensity score matching was employed to control for confounding variables. The propensity score, representing the likelihood of a patient receiving magnesium sulfate, was derived from cox proportional hazards models. Factors included in the propensity score model for matching were determined based on established consensus statements present in the current literature [17]. This model incorporated variables such as age, sex, race, ICU type, Charlson comorbidity index, APACHE III, SAPS II, OASIS, and levels of magnesium.

The matching approach was carried out at a 1:1 ratio utilizing the nearest neighbor technique, applying a caliper width of 0.2 with no substitutions made. The evaluation of variables between the groups was conducted through standardized mean differences (SMD), with values below 0.10 indicating an adequate balance. In the matched cohort dataset, variables that achieved statistical significance (*p* < 0.05) in the univariable analysis were included in multivariable analysis for further adjustment, including heart rate, temperature, systolic blood pressure, magnesium status, mechanical ventilation, myocardial infarct, diuretics, vasodilator, vasopressor, statins, beta-blockers, aspirin, ADP receptor antagonists, diabetes, peripheral vascular disease, creatinine, white blood cells, platelet, hemoglobin, calcium, potassium, sodium, chloride, phosphate and albumin (**Supplementary Table 1**).

#### 2.5.2 Subgroup Analyses

The risk ratio associated with magnesium sulfate use and all-cause mortality in patients with acute heart failure was stratified by age, gender, Charlson comorbidity index, and type of intensive care unit. The likelihood ratio test was employed to assess interactions across subgroups.

#### 2.5.3 Sensitivity Analysis

An analysis of sensitivity utilizing E-values was performed to assess how potential unmeasured or residual confounders might influence the results within our matched cohort [18]. Furthermore, sensitivity analyses were executed on the entire cohort dataset to determine the robustness of the findings obtained from the matched cohort. In the multivariate analysis for adjustment, variables that had a *p*-value of less than 0.05 in the univariate analysis were incorporated. These variables encompassed age, gender, race, type of ICU, Charlson comorbidity index, APACHE III, OASIS, magnesium status, counts of white blood cells and platelets, levels of hemoglobin, blood glucose, potassium, sodium, chloride, magnesium, phosphate, albumin, aspartate aminotransferase, and alanine aminotransferase, resting heart rate, systolic blood pressure, body temperature, diabetes, hypertension, myocardial infarction, peripheral vascular disease, renal disease, along with specific treatments like mechanical ventilation, intravenous diuretics, vasodilators, vasopressors, ACEI/ARB, statins, beta-blockers, aspirin, and ADP receptor antagonists (**Supplementary Table 1**).

Sample size calculation was conducted using PASS 2023 (NCSS Statistical Software, Kaysville, UT, USA). A two-group parallel design was employed to assess whether the proportion for Group 2 (intravenous magnesium sulfate) differs from that of Group 1 (non-intravenous magnesium sulfate). To perform the comparison, a two-sided, two-sample Z-test was utilized, setting the Type I error rate (α) at 0.05. In order to achieve a conditional power of 80% with the current sample sizes, the re-evaluated required sample sizes are N1 = 2527 participants for Group 1 and N2 = 2527 participants for Group 2.

All analyses were performed using R statistical software (version 4.2.2; R Foundation for Statistical Computing, Vienna, Austria) in conjunction with the Free statistical analysis platform (Version 2.0, Beijing, China). A significance threshold of two-sided *p*-values lower than 0.05 was set.

## 3. Results

### 3.1 Patient Selection

**Fig. 1.**
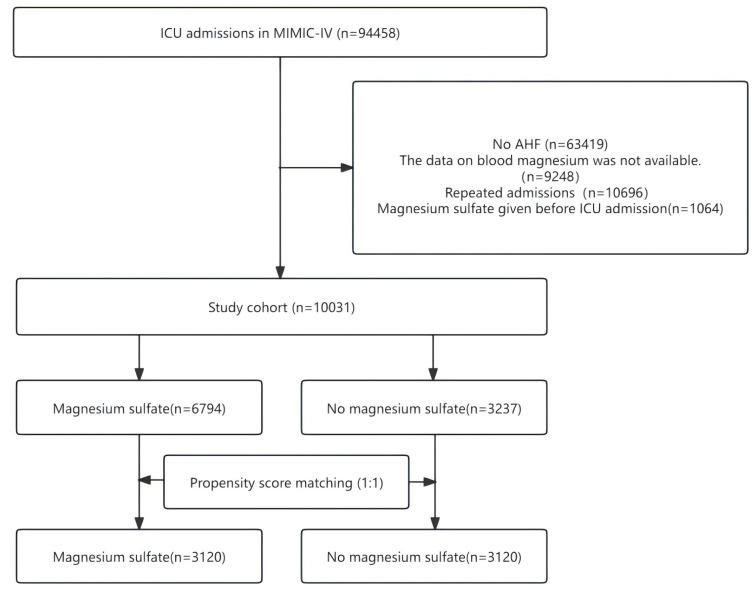
**The flow chart of participant selection**. ICU, intensive care unit; MIMIC-IV, Medical Information Mart for Intensive Care IV; AHF, acute heart failure.

Fig. 1 depicts the procedure for patient selection. The MIMIC IV(3.1) database contains 94,458 ICU hospitalization records. Among them, 63,419 participants were excluded due to non-acute heart failure, 9248 due to missing blood magnesium data, 10,696 due to repeated hospitalization, and 1064 due to magnesium sulfate administration before admission to the ICU. After excluding records that did not meet the inclusion criteria, 10,031 patients were included in the matching cohort, with 6794 (67.7%) receiving intravenous magnesium sulfate while in the ICU. The matched cohort consisted of 6240 patients, 3120 in each group.

Table 1 presents the baseline characteristics prior to and following matching. Within the full cohort, serum magnesium concentrations were markedly reduced in the intravenous magnesium sulfate group when compared to the non-intravenous magnesium sulfate group (*p* < 0.001). The occurrence of hypomagnesemia was notably more prevalent in the magnesium sulfate group (1.32%, 90/6794) in contrast to the non-intravenous group (0.53%, 17/3237) (*p* < 0.001). Patients who received magnesium sulfate were younger and had more hospitalizations in the cardiovascular care unit, had a lower Charlson comorbidity index, and were more likely to receive diuretics via intravenous infusion, vasodilator, or vasopressors. The process of matching enhanced the balance of variables, achieving an absolute standardized mean difference (SMD) of less than 0.10, though some imbalances persisted in variables that were not included in the propensity score matching (Table 1).

**Table 1.** **Baseline characteristics before and after propensity score matching**.

Variables		Before propensity score matching				After propensity score matching			
		All patients (n = 10,031)	No magnesium sulfate (n = 3237)	Magnesium sulfate (n = 6794)	SMD	All patients (n = 6240)	No magnesium sulfate (n = 3120)	Magnesium sulfate (n = 3120)	SMD
Age, y		70.77 ± 13.79	72.24 ± 13.74	70.07 ± 13.76	0.158	72.07 ± 13.40	72.07 ± 13.77	72.06 ± 13.02	0.001
Gender, n (%)					0.012				0.012
	Female	4568 (45.54)	1487 (45.94)	3081 (45.35)		2858 (45.80)	1438 (46.09)	1420 (45.51)	
	Male	5463 (54.46)	1750 (54.06)	3713 (54.65)		3382 (54.20)	1682 (53.91)	1700 (54.49)	
Race, n (%)					0.226				0.033
	White	6826 (68.05)	2259 (69.79)	4567 (67.22)		4340 (69.55)	2189 (70.16)	2151 (68.94)	
	Black	1179 (11.75)	466 (14.4)	713 (10.49)		872 (13.97)	425 (13.62)	447 (14.33)	
	Other	943 (9.40)	294 (9.08)	649 (9.55)		597 (9.57)	288 (9.23)	309 (9.90)	
	Unknown	1083 (10.80)	218 (6.73)	865 (12.73)		431 (6.91)	218 (6.99)	213 (6.83)	
Type of ICU, n (%)					0.380				0.023
	Cardiovascular ICU	4595 (45.81)	1117 (34.51)	3478 (51.19)		2265 (36.30)	1117 (35.8)	1148 (36.79)	
	Medical ICU	2424 (24.17)	1063 (32.84)	1361 (20.03)		1900 (30.45)	964 (30.9)	936 (30.00)	
	Surgical ICU	2203 (21.96)	821 (25.36)	1382 (20.34)		1610 (25.80)	805 (25.8)	805 (25.80)	
	Other	809 (8.06)	236 (7.29)	573 (8.43)		465 (7.45)	234 (7.50)	231 (7.40)	
Anthropometric measure									
	Heart rate	84.34 ± 16.83	82.48 ± 16.46	85.23 ± 16.93	0.165	83.27 ± 16.46	82.72 ± 16.53	83.81 ± 16.37	0.067
	SBP, mmHg	117.06 ± 17.34	119.96 ± 19.00	115.67 ± 16.30	0.244	118.62 ± 18.19	119.90 ± 19.06	117.34 ± 17.18	0.142
	DBP, mmHg	62.42 ± 11.53	62.61 ± 11.89	62.33 ± 11.36	0.027	62.56 ± 11.65	62.72 ± 11.89	62.39 ± 11.40	0.030
	MBP, mmHg	77.49 ± 11.03	77.44 ± 11.91	77.51 ± 10.59	0.006	77.59 ± 11.48	77.52 ± 11.94	77.66 ± 11.01	0.011
	Temperature, °C	36.76 ± 0.49	36.68 ± 0.49	36.80 ± 0.48	0.249	36.73 ± 0.46	36.68 ± 0.49	36.77 ± 0.44	0.185
	Respiratory Rate, insp/min	20.06 ± 3.81	19.97 ± 3.91	20.11 ± 3.77	0.036	20.02 ± 3.80	20.00 ± 3.93	20.05 ± 3.67	0.014
Severity of illness									
	Charlson comorbidity index	7.30 ± 2.55	7.62 ± 2.54	7.15 ± 2.54	0.186	7.58 ± 2.51	7.55 ± 2.52	7.61 ± 2.50	0.022
	APACHE III	50.98 ± 21.60	50.18 ± 19.69	51.36 ± 22.44	0.056	50.21 ± 20.23	50.06 ± 19.72	50.36 ± 20.73	0.015
	SAPS II	39.12 ± 12.94	38.79 ± 12.88	39.28 ± 12.97	0.038	38.72 ± 12.38	38.68 ± 12.92	38.76 ± 11.82	0.006
	OASIS	32.51 ± 8.93	30.94 ± 8.21	33.26 ± 9.16	0.268	31.11 ± 8.35	31.12 ± 8.23	31.10 ± 8.47	0.002
Magnesium status, n (%)					0.084				0.066
	Hypomagnesaemia, n (%)	107 (1.07)	17 (0.53)	90 (1.32)		52 (0.83)	17 (0.54)	35 (1.12)	
	Hypermagnesemia, n (%)	34 (0.34)	11 (0.34)	23 (0.34)		23 (0.37)	10 (0.32)	13 (0.42)	
	Normomagnesaemia, n (%)	9890 (98.59)	3209 (99.14)	6681 (98.34)		6165 (98.80)	3093 (99.13)	3072 (98.46)	
Treatments within ICU admission, n (%)									
	Mechanical ventilation, n (%)	1832 (18.26)	669 (20.67)	1163 (17.12)	0.091	1129 (18.09)	648 (20.77)	481 (15.42)	0.139
	Diuretics, n (%)	4601 (45.87)	1329 (41.06)	3272 (48.16)	0.143	2746 (44.01)	1282 (41.09)	1464 (46.92)	0.118
	Vasopressor, n (%)	2366 (23.59)	443 (13.69)	1923 (28.3)	0.365	1172 (18.78)	435 (13.94)	737 (23.62)	0.250
	Vasodilator, n (%)	2083 (20.77)	362 (11.18)	1721 (25.33)	0.373	1012 (16.22)	352 (11.28)	660 (21.15)	0.270
	Digoxin, n (%)	347 (3.46)	116 (3.58)	231 (3.4)	0.010	211 (3.38)	114 (3.65)	97 (3.11)	0.030
	Statins, n (%)	4596 (45.8)	1181 (36.5)	3415 (50.3)	0.281	2663 (42.7)	1137 (36.4)	1526 (48.9)	0.254
	ACEI/ARB, n (%)	2238 (22.3)	641 (19.8)	1597 (23.5)	0.090	1280 (20.5)	627 (20.1)	653 (20.9)	0.021
	β-blockers, n (%)	4951 (49.4)	1297 (40.1)	3654 (53.8)	0.277	2849 (45.7)	1258 (40.3)	1591 (51)	0.216
	Aspirin, n (%)	4682 (46.7)	1159 (35.8)	3523 (51.9)	0.328	2631 (42.2)	1125 (36.1)	1506 (48.3)	0.249
	ADP receptor antagonists, n (%)	1324 (13.2)	341 (10.5)	983 (14.5)	0.119	724 (11.6)	331 (10.6)	393 (12.6)	0.062
Comorbidities (%)									
	QT prolongation syndromes, n (%)	74 (0.74)	16 (0.49)	58 (0.85)	0.044	44 (0.71)	16 (0.51)	28 (0.9)	0.046
	Hypertension, n (%)	5110 (50.94)	1733 (53.54)	3377 (49.71)	0.077	3293 (52.77)	1678 (53.78)	1615 (51.76)	0.040
	Diabetes, n (%)	4873 (48.58)	1713 (52.92)	3160 (46.51)	0.128	3178 (50.93)	1641 (52.6)	1537 (49.26)	0.067
	Cerebrovascular disease, n (%)	1237 (12.33)	394 (12.17)	843 (12.41)	0.007	781 (12.52)	382 (12.24)	399 (12.79)	0.016
	Myocardial infarct, n (%)	3328 (33.18)	964 (29.78)	2364 (34.8)	0.107	1958 (31.38)	935 (29.97)	1023 (32.79)	0.061
	Chronic pulmonary disease, n (%)	3574 (35.63)	1197 (36.98)	2377 (34.99)	0.042	2312 (37.05)	1146 (36.73)	1166 (37.37)	0.013
	Rheumatic disease, n (%)	421 (4.20)	120 (3.71)	301 (4.43)	0.037	254 (4.07)	116 (3.72)	138 (4.42)	0.036
	Renal disease, n (%)	4068 (40.55)	1637 (50.57)	2431 (35.78)	0.302	2857 (45.79)	1547 (49.58)	1310 (41.99)	0.153
	Severe liver disease, n (%)	268 (2.67)	87 (2.69)	181 (2.66)	0.001	188 (3.01)	83 (2.66)	105 (3.37)	0.041
	Malignant cancer, n (%)	1009 (10.06)	375 (11.58)	634 (9.33)	0.074	724 (11.60)	356 (11.41)	368 (11.79)	0.012
	Peripheral vascular disease, n (%)	1673 (16.68)	500 (15.45)	1173 (17.27)	0.049	1009 (16.17)	463 (14.84)	546 (17.5)	0.072
	Dementia, n (%)	444 (4.43)	149 (4.6)	295 (4.34)	0.013	313 (5.02)	142 (4.55)	171 (5.48)	0.043
Laboratory tests									
	Creatinine, mg/dL	1.50 (1.00, 2.40)	1.70 (1.10, 3.00)	1.40 (1.00, 2.20)	0.251	1.50 (1.10, 2.60)	1.70 (1.10, 2.90)	1.50 (1.00, 2.20)	0.321
	Glucose, mg/dL	135.25 (113.46, 171.00)	133.50 (109.40, 174.20)	135.80 (115.80, 169.70)	0.018	134.50 (111.87, 172.50)	133.67 (109.50, 174.73)	135.00 (114.25, 170.19)	0.020
	White blood cells, K/uL	10.40 (7.60, 14.50)	9.30 (7.10, 13.00)	11.00 (8.00, 15.10)	0.188	9.90 (7.30, 13.80)	9.30 (7.10, 13.00)	10.40 (7.60, 14.60)	0.141
	Platelet, K/uL	210.06 ± 98.55	217.25 ± 100.54	206.63 ± 97.40	0.105	211.98 ± 99.18	217.92 ± 100.66	206.04 ± 97.33	0.118
	Hemoglobin, g/dL	10.59 ± 2.25	10.51 ± 2.17	10.63 ± 2.29	0.052	10.48 ± 2.20	10.54 ± 2.17	10.42 ± 2.22	0.057
	Calcium, mg/dL	8.48 ± 0.65	8.61 ± 0.68	8.42 ± 0.63	0.280	8.53 ± 0.67	8.61 ± 0.68	8.45 ± 0.65	0.237
	Potassium, mEq/L	4.22 ± 0.48	4.28 ± 0.54	4.18 ± 0.44	0.200	4.24 ± 0.50	4.28 ± 0.54	4.19 ± 0.46	0.180
	Sodium, mEq/L	138.09 ± 4.47	138.31 ± 4.56	137.98 ± 4.42	0.074	138.18 ± 4.59	138.30 ± 4.51	138.06 ± 4.67	0.053
	Chloride, mEq/L	102.02 ± 5.80	101.51 ± 5.88	102.27 ± 5.74	0.130	101.81 ± 5.93	101.53 ± 5.82	102.09 ± 6.02	0.096
	Phosphate, mg/dL	3.94 ± 1.28	4.20 ± 1.44	3.81 ± 1.18	0.298	4.01 ± 1.34	4.18 ± 1.43	3.84 ± 1.21	0.260
	Magnesium, mg/dL	2.12 ± 0.41	2.14 ± 0.32	2.11 ± 0.45	0.075	2.13 ± 0.39	2.14 ± 0.31	2.12 ± 0.45	0.030
	Albumin, g/dL	3.3 ± 0.6	3.3 ± 0.5	3.2 ± 0.6	0.194	3.3 ± 0.6	3.3 ± 0.5	3.2 ± 0.6	0.221
	Alanine Aminotransferase, IU/L	33.0 (17.0, 91.0)	32.0 (16.0, 92.0)	34.0 (18.0, 91.0)	0.001	32.0 (16.0, 89.2)	32.0 (16.0, 93.0)	32.0 (17.0, 86.0)	0.029
	Aspartate Aminotransferase, IU/L	48.0 (25.0, 139.0)	45.0 (24.0, 134.0)	50.0 (26.0, 139.8)	0.002	46.0 (25.0, 133.0)	45.0 (24.0, 134.0)	47.0 (25.0, 132.0)	0.005

DBP, Diastolic blood pressure; SBP, Systolic blood pressure; MBP, Mean blood pressure; SMD, standardised mean difference; APACHE, Acute Physiology and Chronic Health Evaluation; SAPS, Simplified Acute Physiology Score; OASIS, Oxford acute severity of illness score; ACEI, angiotensin converting enzyme inhibitor; ARB, angiotensin II receptor blockers; ADP, adenosine diphosphate.

### 3.2 Primary Outcome

**Fig. 2.**
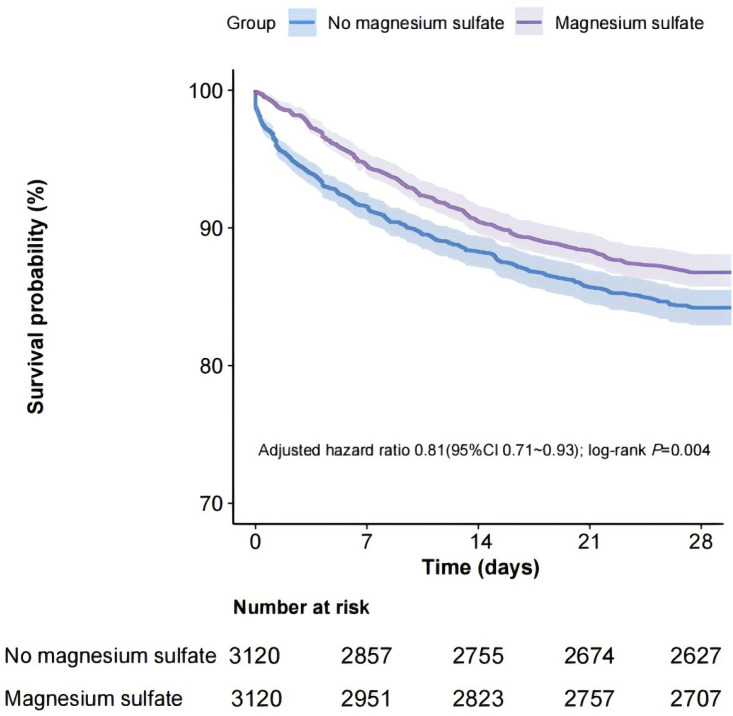
**Kaplan–Meier curve for 28-day all-cause mortality according to magnesium sulfate use in the matched cohort**. CI, confidence interval.

The rate of all-cause mortality over a 28-day period was found to be 13.2% (413/3120) in the group that received magnesium sulfate, compared to 15.8% (493/3120) in the group that did not receive the treatment. In the matched cohort dataset, the variables that demonstrated statistical significance (*p* < 0.05) during the univariate analysis were considered for inclusion in the multivariate analysis to ensure thorough adjustment (**Supplementary Table 1**). Fig. 2 presents the Kaplan–Meier curve, which reflects the all-cause mortality rates over 28 days related to the administration of magnesium sulfate in the matched cohort. Findings from the multivariable analysis indicated an association between the use of magnesium sulfate and a decreased all-cause mortality rate throughout the 28 days (HR, 0.81; 95% CI, 0.71–0.93; *p* = 0.004), corroborated by the results from the univariable analysis (HR, 0.81; 95% CI, 0.71–0.93; *p* = 0.002).

### 3.3 Subgroup Analyses

**Fig. 3.**
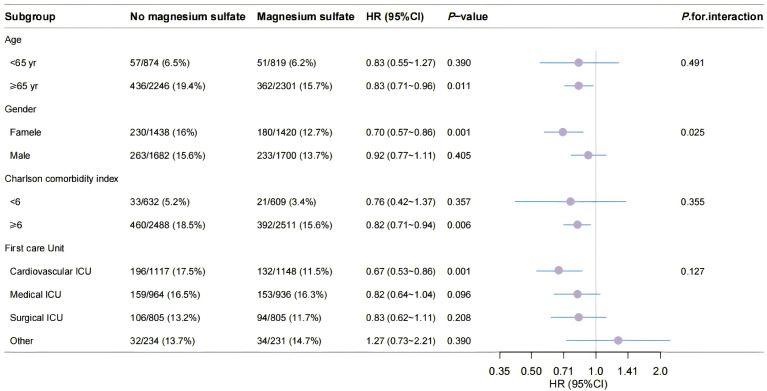
**Subgroup analyses for 28-day all-cause mortality in the matched cohort**. yr, year; HR, hazard ratio.

The findings of subgroup analyses regarding 28-day all-cause mortality within the matched cohort are illustrated in Fig. 3. The analysis revealed a notable interaction between sex and the administration of magnesium sulfate among patient subgroups categorized by gender (*p* for interaction = 0.025). In female patients, the relationship between magnesium sulfate administration and mortality was significantly more pronounced when compared to males (HR, 0.70; 95% CI, 0.57–0.86; *p* = 0.001). The association between magnesium sulfate use and mortality was similar among patient subgroups stratified by age, Charlson score, and intensive care unit type (*p* for interaction >0.05). In all subgroups, patients who received magnesium sulfate had a lower risk of all-cause death than those who did not receive magnesium sulfate. In all subgroups, patients who received intravenous magnesium sulfate exhibited a lower risk of all-cause mortality compared to those who did not receive intravenous magnesium sulfate (HR <1).

### 3.4 Sensitivity Analyses

The E-value suggests that the link between the use of magnesium sulfate and all-cause mortality is strong (E-value: 1.77; upper confidence interval: 1.36). This implies that for the observed relationship between magnesium sulfate administration and overall mortality to be impacted, unmeasured confounders would need to have a risk ratio of at least 1.77. Specifically, only those confounders that exceed a risk ratio of 1.77 would have the potential to alter the findings of the correlation analysis (E-value for point estimate: 1.77; E-value for confidence interval: 1.36) (**Supplementary Fig. 1**). Additionally, we performed sensitivity analyses on the full dataset to assess the robustness of the results obtained from the matched cohort. In the subset that received magnesium sulfate, the mortality rate over 28 days was 13.1% (892/6794), compared to 15.9% (514/3237) in the non-treatment group. The administration of magnesium sulfate was associated with a reduced rate of all-cause mortality at 28 days, as demonstrated by both univariable analysis (HR, 0.80; 95% CI, 0.72–0.90; *p* < 0.001) and multivariable analysis (HR, 0.77; 95% CI, 0.69–0.87; *p* < 0.001) (**Supplementary Table 2**).

### 3.5 Secondary Outcomes

The 90-day mortality rate from all causes in the group receiving magnesium sulfate was noted to be 21.4% (667/3120), whereas the group that did not receive magnesium sulfate recorded a rate of 23.5% (733/3120). In multivariable analysis, the administration of magnesium sulfate was associated with a decreased risk of all-cause mortality at 90 days (HR, 0.88; 95% CI, 0.79–0.99; *p* = 0.029), a result that was further corroborated by univariable analysis (HR, 0.88; 95% CI, 0.80–0.98; *p* = 0.021). Regarding the 365-day all-cause mortality rate, the magnesium sulfate group showed a rate of 32.4% (1010/3120), in contrast to a 36.1% (1125/3120) rate for the group not receiving it. Once more, the use of magnesium sulfate was associated with a reduced 90-day all-cause mortality risk in multivariable analysis (HR, 0.88; 95% CI, 0.80–0.96; *p* = 0.006) and was also validated in univariable analysis (HR, 0.87; 95% CI, 0.80–0.95; *p* = 0.001) (Table 2).

**Table 2.** **The association of magnesium sulfate use with outcomes in the matched cohort**.

Outcomes		No magnesium sulfate (n = 3120)	Magnesium sulfate (n = 3120)	Univariable analysis		Multivariable analysis	
				HR (95% CI)	*p*-value	HR (95% CI)	*p*-value
Primary outcome							
	28-day all-cause mortality, n (%)	493 (15.8%)	413 (13.2%)	0.81 (0.71–0.93)	0.002	0.81 (0.71–0.93)	0.004
Secondary outcomes							
	90-day all-cause mortality, n (%)	733 (23.5)	667 (21.4)	0.88 (0.80–0.98)	0.021	0.88 (0.79–0.99)	0.029
	365-day all-cause mortality, n (%)	1125 (36.1)	1010 (32.4)	0.87 (0.80–0.95)	0.001	0.88 (0.80–0.96)	0.006

## 4. Discussion

In this retrospective cohort study, we found that intravenous magnesium sulfate reduced the risk of 28-day all-cause mortality in patients with acute heart failure in the intensive care unit, and sensitivity analyzes suggested that these results were robust. Our subgroup analysis revealed that this relationship was particularly pronounced in female patients. Furthermore, intravenous magnesium sulfate was linked to a reduction in 90-day and 365-day all-cause mortality.

### 4.1 Relationship With Previous Studies

Research has previously examined the link between magnesium supplementation and the likelihood of all-cause mortality across different populations. A retrospective cohort analysis conducted by Gu *et al*. [17] indicated that the use of intravenous magnesium sulfate significantly decreases mortality rates in critically ill patients suffering from sepsis. In a similar vein, Barbosa* et al*. [19] reported that intravenous magnesium sulfate also diminishes the risk of death among critically ill individuals experiencing acute kidney injury. Although Barbosa *et al*.’s investigation [19] was primarily restricted to a population with low magnesium levels, the findings from Gu *et al*. [17] illustrated that the positive impact of intravenous magnesium sulfate on mortality was not reliant on serum magnesium concentrations. In cases of heart failure, there is a strong correlation between magnesium and the advancement of the disease [20]. Nonetheless, the exploration of the connection between magnesium supplementation and the all-cause mortality risk remains under-researched. A propensity score-matched cohort study by Adamopoulos *et al*. [21] disclosed that in patients diagnosed with chronic heart failure, serum magnesium levels falling below 2 mEq/L were linked to higher cardiovascular mortality. This study, however, did not explore the impact of intravenous magnesium sulfate supplementation on mortality risk and was limited to patients with chronic heart failure. Research by Zhao *et al*. [22] indicated that individuals with higher dietary magnesium intake exhibited a reduced likelihood of developing congestive heart failure. The focus of their investigation was on dietary magnesium supplementation, leaving the relationship between intravenous magnesium supplementation and mortality unaddressed. A systematic review that analyzed serum magnesium levels and heart failure outcomes included 13,539 patients diagnosed with heart failure with reduced ejection fraction (HFrEF) and assessed how serum magnesium levels influenced cardiovascular mortality, all-cause mortality, and cardiovascular morbidity. Among the studies reviewed, hypomagnesemia was recognized as an independent risk factor for cardiovascular death, including instances of sudden cardiac death [23]. Nevertheless, this review failed to assess whether intravenous magnesium sulfate enhances the outcomes for patients with heart failure.

Our research involving individuals experiencing acute heart failure revealed that supplementation with intravenous magnesium sulfate lowered the risk of mortality from all causes in both a matched cohort and the general population. Furthermore, the use of intravenous magnesium sulfate correlated with a decrease in mid-term (90 days) and long-term (365 days) mortality rates. Taken together, these findings reinforce the link between magnesium treatment and improved outcomes for patients suffering from heart failure.

Moreover, our research revealed variations based on gender in how magnesium sulfate supplementation relates to the risk of overall mortality among patients experiencing acute heart failure. In particular, the link between intravenous magnesium sulfate supplementation and all-cause mortality risk seemed to be more significant in female patients. These results are consistent with the findings of Zhang *et al*. [24], who likewise noted that magnesium intake from diet lowers the mortality risk associated with congestive heart failure in women, whereas such a connection was not found in men.

### 4.2 Possible Explanations for Our Findings

Magnesium, the second most commonly found intracellular cation after potassium, is involved in over 600 enzymatic processes, such as energy metabolism and protein synthesis [25]. The role of magnesium ions is significant in myocardial excitation-contraction coupling [26]. Often recognized as a natural opponent to calcium, Mg^2+^ competes with Ca^2+^ for attachment sites on proteins and calcium transporters [7, 27]. The influence of Mg^2+^ on cardiomyocytes largely stems from its effect on calcium mobilization. It binds to calmodulin, troponin C, and parvalbumin, where a reduction in Mg^2+^ concentration can result in alterations to the free Ca^2+^ fraction [28]. Furthermore, Mg^2+^ has the ability to impact the key calcium-transporting proteins within cardiomyocytes, acting as a substrate alongside Adenosine Triphosphate (ATP) for cardiac Ca^2+^-ATPases and altering the affinity of the Na^+^-Ca^2+^ exchanger type 1 (NCX1) for calcium. However, empirical studies investigating the effects of Mg^2+^ on NCX1 and Sarco/Endoplasmic Reticulum Calcium ATPase (SERCA) activity are limited, with most existing data being derived from modeling and in vitro assessments [29, 30]. Regardless, maintaining ideal levels of [Mg^2+^] in cardiac cells is crucial for optimal heart function. Research indicates that magnesium supplementation can mitigate oxidative stress damage and provide protective benefits [31, 32]. Both the disruption of cardiac excitation-contraction coupling and oxidative stress are significant contributors to heart failure. The administration of intravenous magnesium sulfate may potentially affect these physiological mechanisms, thereby offering a positive effect on heart failure patient outcomes.

### 4.3 Implications for Clinical Practice

Magnesium sulfate primarily serves in the treatment of moderate to severe gestational hypertension, eclampsia, hypomagnesemia, torsade de pointes, and pediatric convulsions. Currently, it is not regarded as a standard intervention for acute heart failure. Despite the absence of randomized controlled trials (RCTs) assessing the use of magnesium sulfate specifically for acute heart failure, it was identified last century that a sudden increase in serum magnesium levels after intravenous magnesium therapy can reduce the incidence of ventricular arrhythmias in patients experiencing congestive heart failure [33]. These ventricular arrhythmias significantly contribute to mortality rates in heart failure patients. Findings from the second Leicester Intravenous Magnesium Intervention Trial (LIMIT-2) indicate that intravenous magnesium sulfate is a straightforward, safe, and broadly applicable treatment option. Its effectiveness in lowering early mortality associated with myocardial infarction is similar to that achieved with thrombolytic or antiplatelet therapies, yet it operates independently of these treatments. Myocardial infarction stands as a leading factor in the onset of acute heart failure [34]. This research demonstrates that intravenous magnesium sulfate can reduce short-term, mid-term, and long-term all-cause mortality for patients suffering from acute heart failure, highlighting the necessity for additional prospective RCTs to explore this further.

### 4.4 Study Limitations

The current research possesses several notable limitations. Firstly, due to its design being retrospective and observational, the outcomes may be subject to residual bias and unmeasured confounding variables, even with the use of propensity score matching and multivariable analyses. Nevertheless, the E-value indicates that the findings from the association analysis are substantial. Secondly, establishing a causal connection was not feasible, necessitating additional RCTs. Thirdly, as the severity of patients’ clinical conditions may change over time, this can potentially affect the results. Fourthly, the guidelines for starting and stopping magnesium sulfate treatment were not recorded. Lastly, while the safety profile of magnesium sulfate was not evaluated in this research, it is important to note that adverse effects from magnesium supplementation are infrequent outside of critical care environments.

## 5. Conclusions

Intravenous magnesium sulfate exhibits a statistically significant reduction in 28-day all-cause mortality (HR, 0.81; 95% CI, 0.71–0.93) among critically AHF patients, with sustained benefits observed at 90-day (HR = 0.88) and 365-day (HR = 0.88) follow-up. Notably, female patients demonstrated a more pronounced mortality reduction (HR = 0.70), suggesting sex-specific therapeutic responses. Mechanistically, magnesium’s dual modulation of myocardial calcium kinetics and oxidative stress pathways may underpin its prognostic benefits. While propensity score matching mitigated baseline confounding, the observed associations warrant confirmation through randomized controlled trials to establish causality and guide clinical guideline updates. These findings position magnesium sulfate as a promising adjunctive therapy for improving short and long-term outcomes in high-risk AHF populations.

## Availability of Data and Materials

The datasets generated and analyzed during the current study are available in the MIMIC-IV database, publicly available on the MIMIC-IV website (https://mimic.mit.edu/).

## Supplementary Material

Supplementary material associated with this article can be found, in the online version, at https://doi.org/10.31083/RCM39206.

## References

[1] Bhatia MS, Sharda SC, Attri R, Pannu AK, Dahiya N (2022). Correlation of mortality with Pro-BNP and precipitating factors of acute heart failure in patients presenting to a medical emergency of tertiary care hospital: an observational study from north India. *European Review for Medical and Pharmacological Sciences*.

[2] Sheehan M, Sokoloff L, Reza N (2024). Acute Heart Failure: From The Emergency Department to the Intensive Care Unit. *Cardiology Clinics*.

[3] Bazmpani MA, Papanastasiou CA, Kamperidis V, Zebekakis PE, Karvounis H, Kalogeropoulos AP (2022). Contemporary Data on the Status and Medical Management of Acute Heart Failure. *Current Cardiology Reports*.

[4] Latif A, Ahsan MJ, Lateef N, Kapoor V, Tran A, Abusnina W (2022). Implementation of Multiple Evidence-Based Heart Failure Therapies. *Current Problems in Cardiology*.

[5] Teragawa H, Matsuura H, Chayama K, Oshima T (2002). Mechanisms responsible for vasodilation upon magnesium infusion in vivo: clinical evidence. *Magnesium Research*.

[6] Liao F, Folsom AR, Brancati FL (1998). Is low magnesium concentration a risk factor for coronary heart disease? The Atherosclerosis Risk in Communities (ARIC) Study. *American Heart Journal*.

[7] Chakraborti S, Chakraborti T, Mandal M, Mandal A, Das S, Ghosh S (2002). Protective role of magnesium in cardiovascular diseases: a review. *Molecular and Cellular Biochemistry*.

[8] Tangvoraphonkchai K, Davenport A (2018). Magnesium and Cardiovascular Disease. *Advances in Chronic Kidney Disease*.

[9] Lutsey PL, Alonso A, Michos ED, Loehr LR, Astor BC, Coresh J (2014). Serum magnesium, phosphorus, and calcium are associated with risk of incident heart failure: the Atherosclerosis Risk in Communities (ARIC) Study. *The American Journal of Clinical Nutrition*.

[10] Gottlieb SS (1989). Importance of magnesium in congestive heart failure. *The American Journal of Cardiology*.

[11] Wester PO (1992). Electrolyte balance in heart failure and the role for magnesium ions. *The American Journal of Cardiology*.

[12] Sueta CA, Patterson JH, Adams KF (1995). Antiarrhythmic action of pharmacological administration of magnesium in heart failure: a critical review of new data. *Magnesium Research*.

[13] Späth G (1988). Magnesium in cardiology. A challenge for new studies. *Wiener Medizinische Wochenschrift (1946)*.

[14] Nielsen FH (2024). The Role of Dietary Magnesium in Cardiovascular Disease. *Nutrients*.

[15] Fang X, Wang K, Han D, He X, Wei J, Zhao L (2016). Dietary magnesium intake and the risk of cardiovascular disease, type 2 diabetes, and all-cause mortality: a dose-response meta-analysis of prospective cohort studies. *BMC Medicine*.

[16] von Elm E, Altman DG, Egger M, Pocock SJ, Gøtzsche PC, Vandenbroucke JP (2007). The Strengthening the Reporting of Observational Studies in Epidemiology (STROBE) statement: guidelines for reporting observational studies. *PLoS Medicine*.

[17] Gu WJ, Duan XJ, Liu XZ, Cen Y, Tao LY, Lyu J (2023). Association of magnesium sulfate use with mortality in critically ill patients with sepsis: a retrospective propensity score-matched cohort study. *British Journal of Anaesthesia*.

[18] Fisher DP, Johnson E, Haneuse S, Arterburn D, Coleman KJ, O’Connor PJ (2018). Association Between Bariatric Surgery and Macrovascular Disease Outcomes in Patients With Type 2 Diabetes and Severe Obesity. *JAMA*.

[19] Barbosa EB, Tomasi CD, de Castro Damasio D, Vinhas M, Lichtenfels B, de Luca Francisco V (2016). Effects of magnesium supplementation on the incidence of acute kidney injury in critically ill patients presenting with hypomagnesemia. *Intensive Care Medicine*.

[20] Delva P (2003). Magnesium and heart failure. *Molecular Aspects of Medicine*.

[21] Adamopoulos C, Pitt B, Sui X, Love TE, Zannad F, Ahmed A (2009). Low serum magnesium and cardiovascular mortality in chronic heart failure: a propensity-matched study. *International Journal of Cardiology*.

[22] Zhao D, Chen P, Chen M, Chen L, Wang L (2024). Association of Magnesium Depletion Score with Congestive Heart Failure: Results from the NHANES 2007-2016. *Biological Trace Element Research*.

[23] Voultsos P, Bazmpani MA, Papanastasiou CA, Papadopoulos CE, Efthimiadis G, Karvounis H (2022). Magnesium Disorders and Prognosis in Heart Failure: A Systematic Review. *Cardiology in Review*.

[24] Zhang W, Iso H, Ohira T, Date C, Tamakoshi A (2012). Associations of dietary magnesium intake with mortality from cardiovascular disease: the JACC study. *Atherosclerosis*.

[25] de Baaij JHF, Hoenderop JGJ, Bindels RJM (2015). Magnesium in man: implications for health and disease. *Physiological Reviews*.

[26] Michailova AP, Belik ME, McCulloch AD (2004). Effects of magnesium on cardiac excitation-contraction coupling. *Journal of the American College of Nutrition*.

[27] Iseri LT, French JH (1984). Magnesium: nature’s physiologic calcium blocker. *American Heart Journal*.

[28] Allouche D, Parello J, Sanejouand YH (1999). Ca2+/Mg2+ exchange in parvalbumin and other EF-hand proteins. A theoretical study. *Journal of Molecular Biology*.

[29] Boyman L, Mikhasenko H, Hiller R, Khananshvili D (2009). Kinetic and equilibrium properties of regulatory calcium sensors of NCX1 protein. *The Journal of Biological Chemistry*.

[30] Breukels V, Konijnenberg A, Nabuurs SM, Touw WG, Vuister GW (2011). The second Ca(2+)-binding domain of NCX1 binds Mg2+ with high affinity. *Biochemistry*.

[31] Shi X, Zhu L, Wang S, Zhu W, Li Q, Wei J (2022). Magnesium Hydride Ameliorates Endotoxin-Induced Acute Respiratory Distress Syndrome by Inhibiting Inflammation, Oxidative Stress, and Cell Apoptosis. *Oxidative Medicine and Cellular Longevity*.

[32] Mohammadi H, Shamshirian A, Eslami S, Shamshirian D, Ebrahimzadeh MA (2020). Magnesium Sulfate Attenuates Lethality and Oxidative Damage Induced by Different Models of Hypoxia in Mice. *BioMed Research International*.

[33] Gottlieb SS, Fisher ML, Pressel MD, Patten RD, Weinberg M, Greenberg N (1993). Effects of intravenous magnesium sulfate on arrhythmias in patients with congestive heart failure. *American Heart Journal*.

[34] Woods KL, Fletcher S, Roffe C, Haider Y (1992). Intravenous magnesium sulphate in suspected acute myocardial infarction: results of the second Leicester Intravenous Magnesium Intervention Trial (LIMIT-2). *Lancet*.

