# Supplementary material for: Association Between Intravenous Magnesium Sulfate and All-Cause Mortality in Patients With Acute Heart Failure: A Propensity Score-Matched Cohort Study in MIMIC-IV
Source: Rev Cardiovasc Med. 2025 Sep 24;26(9):39206. doi: 10.31083/RCM39206 (PMC12516736; doi:10.31083/RCM39206)

**Supplementary Table 1. Baseline characteristics before and after propensity score matching.**

|  | Before propensity score matching | | | | After propensity score matching | | | |
| --- | --- | --- | --- | --- | --- | --- | --- | --- |
| Variables | All patients  (n = 10031) | No magnesium sulfate  (n = 3237) | Magnesium sulfate  (n = 6794) | P | All patients  (n = 6240) | No magnesium sulfate  (n = 3120) | Magnesium sulfate  (n = 3120) | P |
| Age, y | 70.77 ± 13.79 | 72.24 ± 13.74 | 70.07 ± 13.76 | **< 0.001** | 72.07 ± 13.40 | 72.07 ± 13.77 | 72.06 ± 13.02 | 0.970 |
| gender, n (%) |  |  |  | **0.58** |  |  |  | 0.647 |
| Female | 4568 (45.54) | 1487 (45.94) | 3081 (45.35) |  | 2858 (45.80) | 1438 (46.09) | 1420 (45.51) |  |
| Male | 5463 (54.46) | 1750 (54.06) | 3713 (54.65) |  | 3382 (54.20) | 1682 (53.91) | 1700 (54.49) |  |
| Race, n (%) |  |  |  | **< 0.001** |  |  |  | 0.64 |
| White | 6826 (68.05) | 2259 (69.79) | 4567 (67.22) |  | 4340 (69.55) | 2189 (70.16) | 2151 (68.94) |  |
| Black | 1179 (11.75) | 466 (14.4) | 713 (10.49) |  | 872 (13.97) | 425 (13.62) | 447 (14.33) |  |
| Other | 943 ( 9.40) | 294 (9.08) | 649 (9.55) |  | 597 ( 9.57) | 288 (9.23) | 309 (9.9) |  |
| Unknown | 1083 (10.80) | 218 (6.73) | 865 (12.73) |  | 431 ( 6.91) | 218 (6.99) | 213 (6.83) |  |
| Type of ICU, n (%) |  |  |  | **< 0.001** |  |  |  | 0.836 |
| Cardiovascular ICU | 4595 (45.81) | 1117 (34.51) | 3478 (51.19) |  | 2265 (36.30) | 1117 (35.8) | 1148 (36.79) |  |
| Medical ICU | 2424 (24.17) | 1063 (32.84) | 1361 (20.03) |  | 1900 (30.45) | 964 (30.9) | 936 (30) |  |
| Surgical ICU | 2203 (21.96) | 821 (25.36) | 1382 (20.34) |  | 1610 (25.80) | 805 (25.8) | 805 (25.8) |  |
| Other | 809 ( 8.06) | 236 (7.29) | 573 (8.43) |  | 465 ( 7.45) | 234 (7.5) | 231 (7.4) |  |
| Anthropometric measure |  |  |  |  |  |  |  |  |
| Heart rate | 84.34 ± 16.83 | 82.48 ± 16.46 | 85.23 ± 16.93 | **< 0.001** | 83.27 ± 16.46 | 82.72 ± 16.53 | 83.81 ± 16.37 | **0.008** |
| Sbp, mmHg | 117.06 ± 17.34 | 119.96 ± 19.00 | 115.67 ± 16.30 | **< 0.001** | 118.62 ± 18.19 | 119.90 ± 19.06 | 117.34 ± 17.18 | **< 0.001** |
| Dbp, mmHg | 62.42 ± 11.53 | 62.61 ± 11.89 | 62.33 ± 11.36 | 0.252 | 62.56 ± 11.65 | 62.72 ± 11.89 | 62.39 ± 11.40 | 0.273 |
| Mbp, mmHg | 77.49 ± 11.03 | 77.44 ± 11.91 | 77.51 ± 10.59 | 0.736 | 77.59 ± 11.48 | 77.52 ± 11.94 | 77.66 ± 11.01 | 0.624 |
| Temperature, °C | 36.76 ± 0.49 | 36.68 ± 0.49 | 36.80 ± 0.48 | **< 0.001** | 36.73 ± 0.46 | 36.68 ± 0.49 | 36.77 ± 0.44 | **< 0.001** |
| Respiratory Rate, insp/min | 20.06 ± 3.81 | 19.97 ± 3.91 | 20.11 ± 3.77 | 0.086 | 20.02 ± 3.80 | 20.00 ± 3.93 | 20.05 ± 3.67 | 0.572 |
| Severity of illness |  |  |  |  |  |  |  |  |
| Charlson comorbidity index | 7.30 ± 2.55 | 7.62 ± 2.54 | 7.1 ± 2.5 | **< 0.001** | 7.58 ± 2.51 | 7.55 ± 2.52 | 7.61 ± 2.50 | 0.383 |
| APACHE III | 50.98 ± 21.60 | 50.18 ± 19.69 | 51.4 ± 22.4 | **0.011** | 50.21 ± 20.23 | 50.06 ± 19.72 | 50.36 ± 20.73 | 0.567 |
| SAPS Ⅱ | 39.12 ± 12.94 | 38.79 ± 12.88 | 39.3 ± 13.0 | 0.079 | 38.72 ± 12.38 | 38.68 ± 12.92 | 38.76 ± 11.82 | 0.798 |
| OASIS | 32.51 ± 8.93 | 30.94 ± 8.21 | 33.3 ± 9.2 | **< 0.001** | 31.11 ± 8.35 | 31.12 ± 8.23 | 31.10 ± 8.47 | 0.950 |
| Magnesium status, n (%) |  |  |  | 0.001 |  |  |  | 0.035 |
| Hypomagnesaemia, n (%) | 107 ( 1.07) | 17 (0.53) | 90 (1.3) |  | 52 ( 0.83) | 17 (0.54) | 35 (1.12) |  |
| Hypermagnesemia, n (%) | 34 ( 0.34) | 11 (0.34) | 23 (0.3) |  | 23 ( 0.37) | 10 (0.32) | 13 (0.42) |  |
| Normomagnesaemia, n (%) | 9890 (98.59) | 3209 (99.14) | 6881（98.3） |  | 6165 (98.80) | 3093 (99.13) | 3072 (98.46) |  |
| Treatments within ICU admission, n (%) |  |  |  |  |  |  |  |  |
| Mechanical ventilation, n (%) | 1832 (18.26) | 669 (20.67) | 1163 (17.1) | **< 0.001** | 1129 (18.09) | 648 (20.77) | 481 (15.42) | **< 0.001** |
| Diuretics, n (%) | 4601 (45.87) | 1329 (41.06) | 3272 (48.2) | **< 0.001** | 2746 (44.01) | 1282 (41.09) | 1464 (46.92) | **< 0.001** |
| Vasopressor, n (%) | 2366 (23.59) | 443 (13.69) | 1923 (28.3) | **< 0.001** | 1172 (18.78) | 435 (13.94) | 737 (23.62) | **< 0.001** |
| Vasodilator, n (%) | 2083 (20.77) | 362 (11.18) | 1721 (25.3) | **< 0.001** | 1012 (16.22) | 352 (11.28) | 660 (21.15) | **< 0.001** |
| Digoxin, n (%) | 347 ( 3.46) | 116 (3.58) | 231 (3.4) | 0.638 | 211 ( 3.38) | 114 (3.65) | 97 (3.11) | 0.234 |
| Statins, n (%) | 4596 (45.8) | 1181 (36.5) | 3415 (50.3) | < 0.001 | 2663 (42.7) | 1137 (36.4) | 1526 (48.9) | **< 0.001** |
| ACEI, n (%) | 2238 (22.3) | 641 (19.8) | 1597 (23.5) | < 0.001 | 1280 (20.5) | 627 (20.1) | 653 (20.9) | 0.415 |
| β-blockers, n (%) | 4951 (49.4) | 1297 (40.1) | 3654 (53.8) | < 0.001 | 2849 (45.7) | 1258 (40.3) | 1591 (51) | **< 0.001** |
| Aspirin, n (%) | 4682 (46.7) | 1159 (35.8) | 3523 (51.9) | < 0.001 | 2631 (42.2) | 1125 (36.1) | 1506 (48.3) | **< 0.001** |
| ADP receptor antagonists, n (%) | 1324 (13.2) | 341 (10.5) | 983 (14.5) | < 0.001 | 724 (11.6) | 331 (10.6) | 393 (12.6) | **0.014** |
| Comorbidities (%) |  |  |  |  |  |  |  |  |
| QT prolongation syndromes, n (%) | 74 ( 0.74) | 16 (0.49) | 58 (0.9) | 0.049 | 44 ( 0.71) | 16 (0.51) | 28 (0.9) | 0.069 |
| Hypertension, n (%) | 5110 (50.94) | 1733 (53.54) | 3377 (49.7) | **< 0.001** | 3293 (52.77) | 1678 (53.78) | 1615 (51.76) | 0.11 |
| Diabetes, n (%) | 4873 (48.58) | 1713 (52.92) | 3160 (46.5) | **< 0.001** | 3178 (50.93) | 1641 (52.6) | 1537 (49.26) | **0.008** |
| Cerebrovascular disease, n (%) | 1237 (12.33) | 394 (12.17) | 843 (12.4) | 0.737 | 781 (12.52) | 382 (12.24) | 399 (12.79) | 0.515 |
| Myocardial infarct, n (%) | 3328 (33.18) | 964 (29.78) | 2364 (34.8) | **< 0.001** | 1958 (31.38) | 935 (29.97) | 1023 (32.79) | **0.016** |
| Chronic pulmonary disease, n (%) | 3574 (35.63) | 1197 (36.98) | 2377 (35) | 0.051 | 2312 (37.05) | 1146 (36.73) | 1166 (37.37) | 0.6 |
| Rheumatic disease, n (%) | 421 ( 4.20) | 120 (3.71) | 301 (4.43) | 0.091 | 254 ( 4.07) | 116 (3.72) | 138 (4.42) | 0.159 |
| Renal disease, n (%) | 4068 (40.55) | 1637 (50.57) | 2431 (35.78) | **< 0.001** | 2857 (45.79) | 1547 (49.58) | 1310 (41.99) | **< 0.001** |
| Severe liver disease, n (%) | 268 ( 2.67) | 87 (2.69) | 181 (2.66) | 0.945 | 188 ( 3.01) | 83 (2.66) | 105 (3.37) | 0.103 |
| Malignant cancer, n (%) | 1009 (10.06) | 375 (11.58) | 634 (9.33) | **< 0.001** | 724 (11.60) | 356 (11.41) | 368 (11.79) | 0.635 |
| Peripheral vascular disease, n (%) | 1673 (16.68) | 500 (15.45) | 1173 (17.27) | **0.022** | 1009 (16.17) | 463 (14.84) | 546 (17.5) | 0.004 |
| Dementia, n (%) | 444 ( 4.43) | 149 (4.6) | 295 (4.34) | 0.552 | 313 ( 5.02) | 142 (4.55) | 171 (5.48) | 0.093 |
| Laboratory tests |  |  |  |  |  |  |  |  |
| Creatinine, mg/dL | 1.50 (1.00, 2.40) | 1.70 (1.10, 3.00) | 1.40 (1.00, 2.20) | **< 0.001** | 1.50 (1.10, 2.60) | 1.70 (1.10, 2.90) | 1.50 (1.00, 2.20) | **< 0.001** |
| Glucose, mg/dL | 135.25(113.46, 171.00) | 133.50(109.40, 174.20) | 135.80(115.80, 169.70) | **0.002** | 134.50 (111.87, 172.50) | 133.67 (109.50, 174.73) | 135.00 (114.25, 170.19) | 0.107 |
| White blood cells, K/uL | 10.40 (7.60, 14.50) | 9.30 (7.10, 13.00) | 11.00 (8.00, 15.10) | **< 0.001** | 9.90 (7.30, 13.80) | 9.30 (7.10, 13.00) | 10.40 (7.60, 14.60) | **< 0.001** |
| Platelet, K/uL | 210.06 ± 98.55 | 217.25 ± 100.54 | 206.63 ± 97.40 | **< 0.001** | 211.98 ± 99.18 | 217.92 ± 100.66 | 206.04 ± 97.33 | **< 0.001** |
| Hemoglobin, g/dL | 10.59 ± 2.25 | 10.51 ± 2.17 | 10.63 ± 2.29 | **0.014** | 10.48 ± 2.20 | 10.54 ± 2.17 | 10.42 ± 2.22 | **0.025** |
| Calcium, mg/dL | 8.48 ± 0.65 | 8.61 ± 0.68 | 8.42 ± 0.63 | **< 0.001** | 8.53 ± 0.67 | 8.61 ± 0.68 | 8.45 ± 0.65 | **< 0.001** |
| Potassium, mEq/L | 4.22 ± 0.48 | 4.28 ± 0.54 | 4.18 ± 0.44 | **< 0.001** | 4.24 ± 0.50 | 4.28 ± 0.54 | 4.19 ± 0.46 | **< 0.001** |
| Sodium, mEq/L | 138.09 ± 4.47 | 138.31 ± 4.56 | 137.98 ± 4.42 | **< 0.001** | 138.18 ± 4.59 | 138.30 ± 4.51 | 138.06 ± 4.67 | **0.035** |
| Chloride, mEq/L | 102.02 ± 5.80 | 101.51 ± 5.88 | 102.27 ± 5.74 | **< 0.001** | 101.81 ± 5.93 | 101.53 ± 5.82 | 102.09 ± 6.02 | **< 0.001** |
| Phosphate, mg/dL | 3.94 ± 1.28 | 4.20 ± 1.44 | 3.81 ± 1.18 | **< 0.001** | 4.01 ± 1.34 | 4.18 ± 1.43 | 3.84 ± 1.21 | **< 0.001** |
| Magnesium, mg/dL | 2.12 ± 0.41 | 2.14 ± 0.32 | 2.11 ± 0.45 | **< 0.001** | 2.13 ± 0.39 | 2.14 ± 0.31 | 2.12 ± 0.45 | 0.243 |
| Albumin, g/dL | 3.3 ± 0.6 | 3.3 ± 0.5 | 3.2 ± 0.6 | < 0.001 | 3.3 ± 0.6 | 3.3 ± 0.5 | 3.2 ± 0.6 | **< 0.001** |
| Alanine Aminotransferase, IU/L | 33.0 (17.0, 91.0) | 32.0 (16.0, 92.0) | 34.0 (18.0, 91.0) | < 0.001 | 32.0 (16.0, 89.2) | 32.0 (16.0, 93.0) | 32.0 (17.0, 86.0) | 0.256 |
| Aspartate Aminotransferase, IU/L | 48.0 (25.0, 139.0) | 45.0 (24.0, 134.0) | 50.0(26.0, 139.8) | 0.004 | 46.0(25.0, 133.0) | 45.0 (24.0, 134.0) | 47.0 (25.0, 132.0) | 0.359 |

DBP, Diastolic blood pressure; SBP, Systolic blood pressure; MBP, Mean blood pressure; APACHE, Acute Physiology and Chronic Health Evaluation; SAPS, Simplified Acute Physiology Score; OASIS, Oxford acute severity of illness score

**Supplementary Table 2. The association of magnesium sulfate use with outcomes in the entire cohort.**

| Outcomes | No magnesium sulfate  (n = 3237) | Magnesium sulfate  (n = 6794) | Univariable analysis | | Multivariable analysis | |
| --- | --- | --- | --- | --- | --- | --- |
|  |  |  | HR (95% CI) | *P*-value | HR (95% CI) | *P*-value |
| Primary outcome |  |  |  |  |  |  |
| 28-day all-cause mortality, n (%) | 514(15.9%) | 892(13.1%) | 0.80(0.72-0.90) | <0.001 | 0.77(0.69-0.87) | <0.001 |

CI, confidence interval; HR, hazard ratio

**Supplementary Fig. 1. Bias plot of confounding relative risks.**


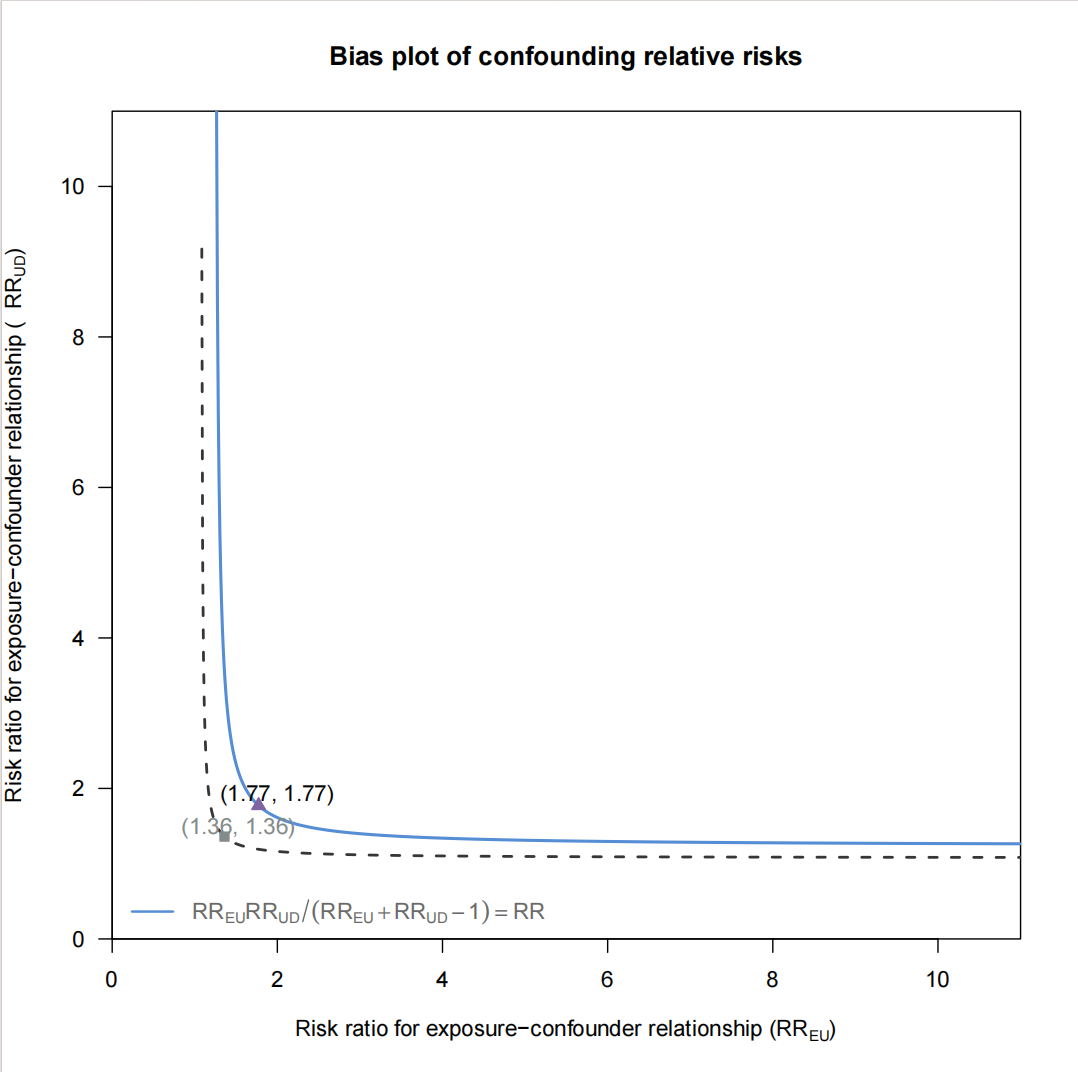

Supplement: Supplementary file 1 [file 2153-8174-26-9-39206-s1.docx]
